# Genome-wide association study of quality traits and starch pasting properties of maize kernels

**DOI:** 10.1186/s12864-022-09031-4

**Published:** 2023-02-02

**Authors:** Xinmei Guo, Zhaopeng Ge, Ming Wang, Meiai Zhao, Yuhe Pei, Xiyun Song

**Affiliations:** 1 College of Agronomy, Qingdao Agricultural University, Qingdao, 266109 China; 2 College of Life Sciences, Qingdao Agricultural University, Qingdao, 266109 China

**Keywords:** Maize, Starch, Pasting properties, Genome-wide association study (GWAS)

## Abstract

**Background:**

Starch are the main nutritional components of maize (*Zea mays L*.), and starch pasting properties are widely used as essential indicators for quality estimation. Based on the previous studies, various genes related to pasting properties have been identified in maize. However, the loci underlying variations in starch pasting properties in maize inbred lines remain to be identified.

**Results:**

To investigate the genetic architecture of these traits, the starch pasting properties were examined based on 292 maize inbred lines, which were genotyped with the MaizeSNP50 BeadChip composed of 55,126 evenly spaced, random SNPs. A genome-wide association study (GWAS) implemented in the software package FarmCPU was employed to identify genomic loci for the starch pasting properties. 48 SNPs were found to be associated with pasting properties. Moreover, 37 candidate genes were correlated with pasting properties. Among the candidate genes, GRMZM2G143646 and GRMZM2G166407 were associated with breakdown and final viscosity significantly, and both genes encode PPR (Pentatricopeptide repeat) protein. We used GWAS to explore candidate genes of maize starch pasting properties in this study. The identified candidate genes will be useful for further understanding of the genetic architecture of starch pasting properties in maize.

**Conclusion:**

This study showed a complex regulation network about maize quality trait and starch pasting properties. It may provide some useful markers for marker assisted selection and a basis for cloning the genes behind these SNPs.

## Background

Maize (*Zea mays L.*), one of the most fundamental crops in the world, plays a crucial role in food, feed and industrial production. The natural population of maize shows abundant phenotypic variation and genotypic variation, offering great convenience for studying the relationship between genotype and phenotypic diversity [1]. Determining the allelic variation of important agronomic traits not only helps to analyze the genetic basis of agronomic traits, but also provides effective gene resources and molecular markers for marker assisted selection (MAS) [2].

Developments in association analysis have heightened the need for analyzing the genetic basis of complex quantitative characters [3]. Association analysis based on a natural population and linkage disequilibrium (LD) can directly identify phenotypic variation-related genes by combining the genetic variation of target traits with genetic polymorphism [4, 5]. A wide range of genetic materials can be simultaneously used to examine the associated sites and alleles of most QTL (Quantitative Trait Locus), not limited by the traditional “two-parent range”. The LD attenuates and exists within a very short distance after many reorganizations, which ensures higher location accuracy [6]. With the development of high-throughput sequencing and other biological technology, GWAS have been verified to be a useful approach for identifying genes, alleles or haplotypes related to a certain agronomic traits under complex environments, which is based on the linkage disequilibrium (LD) resulting from the association of target trait and haplotype loci. GWAS has been widely used in maize genetics, which provides many opportunities for further understanding the genetic basis for controlling the occurrence of complex quantitative characters in maize. Liu et al. (2016) identified 4 starch content related SNPs in chromosomes 1, 2, 5, and 77 starch synthesis related genes by using 263 maize inbred lines [7]. According to genome-wide association study (GWAS) based on genotyping of a natural population, a significant SNP for starch content within the ORF region of GRMZM5G852704_T01 colocalized with QTL Qsta9.1 which located in a 1.7 Mb interval on chromosome 9 [8]. Xu et al. (2018) identified 60 quantitative trait nucleotides (QTNs) for starch pasting properties through GWAS for seven pasting properties of maize starch with a panel of 230 inbred lines and 145,232 SNPs [9].

Starch is a polymeric carbohydrate consisting of numerous glucose units joined by glycosidic bonds called polymers. This polysaccharide is produced by most green plants as energy storage. Plants produce starch by first converting glucose 1-phosphate to ADP-glucose using the enzyme glucose-1-phosphate adenylyltransferase. This step requires energy in the form of ATP. The enzyme starch synthase then adds the ADP-glucose via a 1,4-alpha glycosidic bond to a growing chain of glucose residues, liberating ADP and creating amylose. The ADP-glucose is almost certainly added to the non-reducing end of the amylose polymer, as the UDP-glucose is added to the non-reducing end of glycogen during glycogen synthesis [10]. Moreover, many genes have been found to contribute to starch biosynthesis in maize, and are regulated by a complex regulation network [11].

Starch pasting properties are a critical index for measuring the quality of starch and have an important effect on the application and processing of starch. Therefore, understanding the pasting properties of starch is an important basis for its application [9]. The peak viscosity of starch is determined by the friction between starch granules after water swelling and the increase in viscosity, which reflects the expansibility of starch. The trough viscosity is due to the bursting of starch granules after the expansibility reaches its limit, reflecting the shear resistance of starch at high temperatures. The final viscosity is due to the further increase in viscosity caused by the movement of water molecules surrounding in amylose and amylopectin; this property reflects the hardness of starch at room temperature. Breakdown represents the change in the stability, reflecting the shear resistance of starch at high temperatures. Setback reflects the aging degree of starch. The pasting properties of starch are closely related to the molecular size of amylose and the branching chain length of amylopectin [12].

At present, the study of maize starch is mainly focused on the analysis and evaluation of applied quality, and traditional QTL mapping is used to locate related genes [13, 14]. However, the mapping interval is relatively large. To date, few works have performed a GWAS of starch pasting properties and discovered candidate genes. In this study, a genome-wide association study was performed based on a MaizeSNP50 BeadChip composed of 55,126 and the phenotypic data of 292 maize inbred lines. The aims of this study were to detect pasting properties related genes in maize, and to provide an important theoretical basis for maize quality breeding.

## Results

### Phenotypic variations analysis and genome-wide association study of quality traits

The quality traits in maize are under the control of many factors. In this study, the statistical results of the phenotype of quality traits are listed in Tables 1 and 2. In the four environments, the average protein contents were 11.52%, 11.01%, 11.87%, and 11.34%. The average starch contents were 70.46%, 71.54%, 70.24%, and 70.63%. The average oil contents were 4.54%, 4.35%, 4.35% and 4.35%. The data pertaining to each trait approximately followed a normal distribution, and the absolute values of the kurtosis and skewness among these environments were less than 1; thus, the phenotypic data were suitable for GWAS and further analysis. In the four environments, there was a significant positive correlation between protein content and oil content, except at Luoyang; starch content showed a significant negative correlation with protein content and oil content. The heritability of protein, starch and oil content were 82.73%, 85.82% and 80.69%, respectively.

**Table 1:** Statistical analysis of maize quality traits in different environments

Trait	Environment	CV%	Mean ± SD	Variance	Kurtosis	Skewness	H^2^(%)
Protein	2015LY	8.13	11.52 ± 0.937	0.878	0.221	-0.368	82.73
	2015QZ	9.88	11.01 ± 1.088	1.184	0.36	0.096	
	2016JZ	9.10	11.87 ± 1.080	1.166	0.144	-0.18	
	2017JZ	8.87	11.34 ± 1.006	1.011	0.325	0.588	
Starch	2015LY	1.87	70.46 ± 1.316	1.733	-0.28	0.136	85.82
	2015QZ	1.64	71.54 ± 1.172	1.373	-0.518	0.731	
	2016JZ	1.60	70.24 ± 1.124	1.262	0.3	0.857	
	2017JZ	1.58	70.63 ± 1.117	1.247	0.974	-0.22	
Oil	2015LY	9.52	4.54 ± 0.432	0.186	0.372	0.734	80.69
	2015QZ	12.55	4.35 ± 0.546	0.298	0.298	0.871	
	2016JZ	12.79	4.47 ± 0.572	0.327	0.129	0.161	
	2017JZ	11.87	4.70 ± 0.558	0.334	0.621	0.851	

*SD* Standard deviation, *CV* Coefficient of variation

**Table 2:** Correlation analysis of maize quality traits in different environments

Environment	Trait	Protein	Starch	oil
2015LY	Protein	1		
	Starch	-0.333^**^	1	
	Oil	0.089	-0.390^**^	1
2015QZ	Protein	1		
	Starch	-0.494^**^	1	
	Oil	0.314^**^	-0.711^**^	1
2016JZ	Protein	1		
	Starch	-0.411^**^	1	
	Oil	0.243^**^	-0.278^**^	1
2017JZ	Protein	1		
	Starch	-0.614^**^	1	
	Oil	0.225^**^	-0.739^**^	1

^**^significant at *p* < 0.01; *significant at *p* < 0.05

**Fig. 1:**
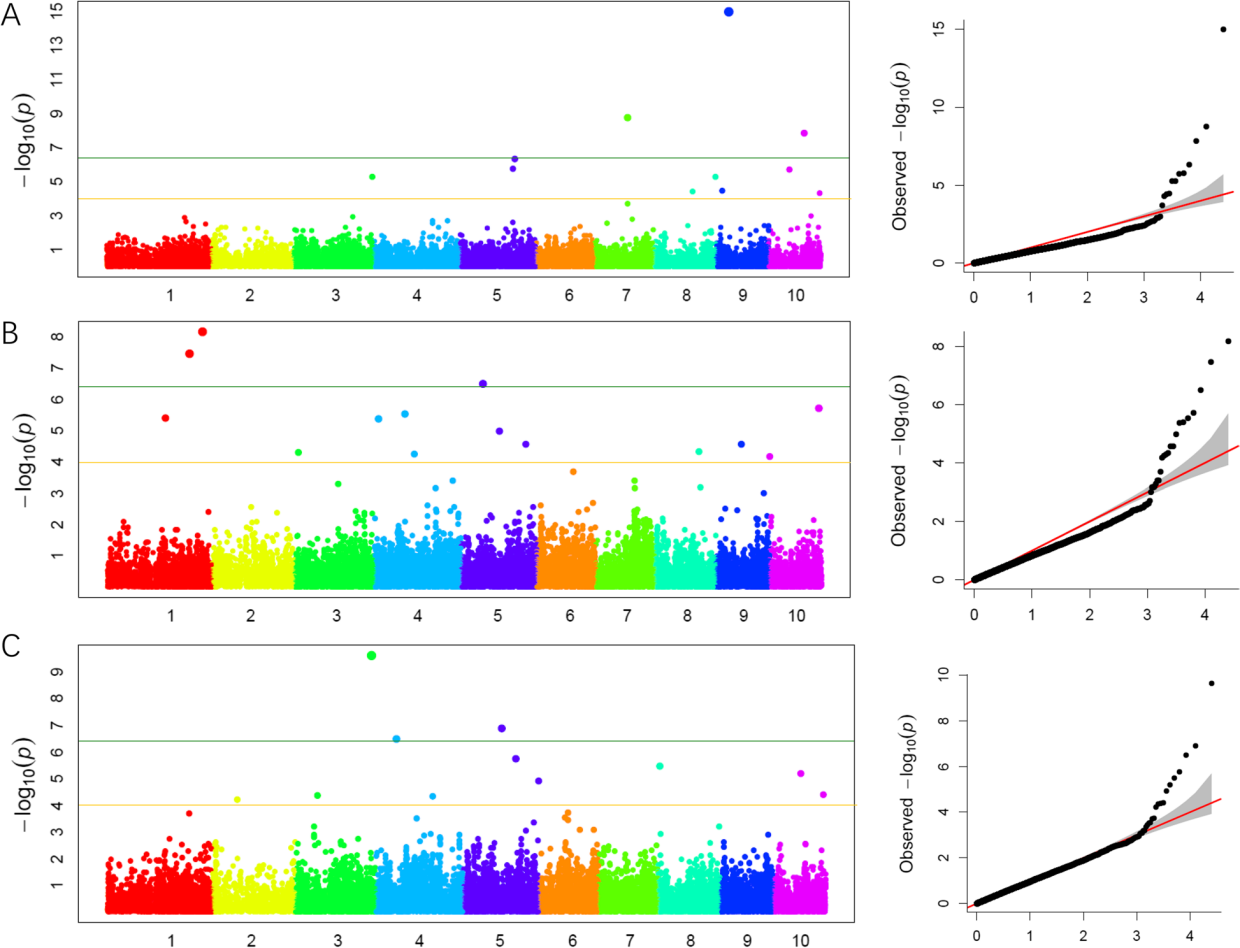
manhattan polt and Q-Q polt by genome-wide association study. **A** protein content; **B** starch content; **C** oil content

In order to find the quality traits related SNPs (Single Nucleotide Polymorphism), the genotype data of the 25,331 SNPs and the phenotypic data of the 292 maize inbred lines were used for genome-wide association study. The analysis identified 26 SNPs at the *P* < 10^–4^ level, based on the FarmCPU methods (Fig. 1) [15]. In the four different environments, 8, 11 and 7 SNPs were identified to correlate to protein, starch and oil content, respectively. PZE_106054189 at Bin6.04 detected in 2015Luoyang and 2016Jiaozhou was correlated with starch content. PZE_108135907 and PZE_109032161 correlated with protein and starch content were detected at Bin8.09 and Bin9.03, respectively. PZE_105086878, PZE_106067078, SYN3414 and PZE_106054189 correlated with starch and oil content were detected at Bin5.04, Bin6.01 and Bin6.04, respectively (Table 3).

**Table 3:** The SNPs associated with quality traits (*P* < 10^–4^)

Trait	SNP	Chr	Genotype	Bin	Position	*P*-value	Environment
Protein	PZE_103041051	3	G/A	3.04	36,763,501	9.01E-10	2015LY
	SYN33251	4	C/T	4.03	16,339,439	1.77E-09	2015QZ
	PZE_106018579	6	G/T	6.01	37,197,726	1.76E-05	2015QZ
	SYN17423	8	G/A	8.05	134,818,226	3.46E-10	2015QZ
	PZE_108135907	8	C/G	8.09	174,418,959	5.62E-06	2017JZ
	PZE_109019092	9	A/G	9.02	19,190,493	3.37E-05	2017JZ
	PZE_109032161	9	C/G	9.03	37,880,373	1.05E-15	2017JZ
	SYN11748	10	C/T	10.07	146,025,814	5.02E-05	2017JZ
Starch	PZE_101130213	1	A/C	1.05	166,556,661	4.11E-06	2017JZ
	PZE_104013159	4	A/G	4.02	11,329,078	4.29E-06	2017JZ
	PZE_105086878	5	A/T	5.04	110,514,145	1.05E-05	2017JZ
	SYN3414	6	G/A	6.01	9,182,861	1.72E-05	2015QZ
	PZE_106054189	6	T/C	6.04	105,019,334	2.59E-05	2016JZ
	PZE_106067078	6	A/G	6.04	119,903,632	5.39E-07	2015QZ
	PZE_106054189	6	T/C	6.04	105,019,334	6.92E-06	2015LY
	PZE_107065016	7	C/G	7.02	116,395,949	1.58E-06	2015LY
	PZE_108005661	8	C/T	8.01	5,774,110	2.18E-05	2015QZ
	PZE_108028552	8	G/C	8.03	31,305,636	7.64E-06	2015LY
	PZE_109032161	9	C/G	9.03	37,880,373	1.55E-05	2015LY
Oil	SYN38947	1	A/G	1.11	298,011,612	9.49E-05	2015QZ
	PZE_104087424	4	G/T	4.06	161,347,637	4.54E-05	2017JZ
	PZE_105086878	5	A/T	5.04	110,514,145	1.30E-07	2017JZ
	SYN3414	6	G/A	6.01	9,182,861	1.05E-06	2015QZ
	PZE_106054189	6	T/C	6.04	105,019,334	2.59E-05	2016JZ
	PZE_106067078	6	A/G	6.04	119,903,632	1.74E-05	2015QZ
	PZE_108091085	8	G/T	8.06	146,812,014	2.18E-05	2015QZ

### Genome-wide association study of starch content

In order to identify the SNPs related to starch content, the genome-wide association study was carried out through the phenotype data from four different environments. The result showed that 37 SNPs were related to starch content under four environments. 9 and 8 SNPs were identified in 2015 at Luoyang and Qingzhou respectively. 6 and 14 SNPs were identified at Jiaozhou in 2016 and 2017 respectively. In addition, the two SNPs, PZE_108135907 and PZE_109032161, were both detected to be related to starch content and protein content. Based on the gene annotation of MaizeGDB database [16], the identified SNPs that related to starch content were related to various metabolism pathways or signaling pathways.

### Phenotypic variations analysis and genome-wide association study of starch pasting properties

The statistical results concerning the phenotype of starch pasting properties are listed in Tables 4 and 5. The data for each trait approximately follow a normal distribution, and the absolute values of the kurtosis and skewness among these environments were less than 1. In the four environments, significant positive correlations were observed between any two parameters among PV, TV, BD, FV, and SB; PT was positively correlated with PTP; and BD was negatively correlated with PT and PTP. The heritability values of PV, TV, BD, FV, SB, PT and PTP were 87.98%, 82.14%, 80.45%, 87.98%, 87.56%, 80.24% and 89.43%, respectively.

**Table 4:** Statistical analysis of pasting properties of maize kernels in different environments

Trait	Environment	CV%	Mean ± SD	Variance	Kurtosis	Skewness	H2(%)
PV	2015LY	13.98	2325.49 ± 325.14	105,716.02	0.109	0.468	87.98
	2015QZ	13.2	2033.96 ± 268.43	72,054.66	0.574	-0.073	
	2016JZ	8.97	2635.83 ± 236.53	55,946.44	0.1	0.317	
	2017JZ	7.92	2275.67 ± 180.17	32,461.23	0.002	0.652	
TV	2015LY	14.06	1640.07 ± 230.62	53,185.58	0.422	0.724	82.14
	2015QZ	13.98	1647.36 ± 230.23	53,005.85	-0.651	0.547	
	2016JZ	13.03	1898.32 ± 247.36	61,186.97	-0.066	0.375	
	2017JZ	9.82	1544.95 ± 151.66	23,000.76	-0.456	0.096	
BD	2015LY	14.23	791.43 ± 112.62	12,683.26	0.276	0.336	80.45
	2015QZ	14.58	786.6 ± 114.65	13,144.62	0.581	-0.651	
	2016JZ	14.07	797.51 ± 112.21	12,591.08	0.312	0.067	
	2017JZ	14.8	780.72 ± 115.53	13,347.18	0.698	0.969	
FV	2015LY	13.98	4504.36 ± 629.51	396,282.84	0.038	-0.041	87.98
	2015QZ	14.87	4961.66 ± 737.84	544,407.87	-0.185	-0.245	
	2016JZ	15	4754.45 ± 713.88	509,624.65	-0.618	0.143	
	2017JZ	14.95	4746.99 ± 709.86	503,901.22	-0.423	-0.199	
SB	2015LY	15.62	2900.29 ± 452.93	205,145.58	-0.357	-0.013	87.56
	2015QZ	14.46	2714.3 ± 392.6	154,134.76	0.354	-0.137	
	2016JZ	16.74	2856.12 ± 478.18	228,656.11	-0.656	0.023	
	2017JZ	14.65	2202.04 ± 322.58	104,057.86	-0.434	-0.464	
PT	2015LY	7.84	5.1 ± 0.4	0.16	0.828	0.415	80.24
	2015QZ	13.5	4.74 ± 0.64	0.42	0.484	0.684	
	2016JZ	7.68	5.47 ± 0.42	0.177	-0.354	0.044	
	2017JZ	8.08	5.2 ± 0.42	0.18	0.181	0.46	
PTP	2015LY	2.93	79.17 ± 2.33	5.42	-0.986	-0.007	89.43
	2015QZ	2.14	76.65 ± 1.64	2.71	0.062	0.853	
	2016JZ	4.24	80.03 ± 3.39	11.51	-0.088	0.312	
	2017JZ	4.93	78.15 ± 3.85	14.84	-0.308	0.4	

*PV* Peak viscosity, *TV* Trough viscosity, *FV* Final viscosity, *SB* Setback (FV − TV), *PT* Peak time, *PTP* Pasting temperature

**Table 5:** Correlation analysis of pasting properties in different environments

Environment	Trait	PV	TV	BD	FV	SB	PT	PTP
2015QZ	PV	1						
	TV	0.825^**^	1					
	BD	0.784^**^	0.295^**^	1				
	FV	0.785^**^	0.932^**^	0.302^**^	1			
	SB	0.703^**^	0.822^**^	0.284^**^	0.972^**^	1		
	PT	-0.193^*^	0.188^*^	-0.532^**^	0.156	0.124	1	
	PTP	-0.206^*^	0.157	-0.500^**^	0.257^**^	0.300^**^	0.590^**^	1
2015LY	PV	1						
	TV	0.920^**^	1					
	BD	0.856^**^	0.586^**^	1				
	FV	0.814^**^	0.893^**^	0.506^**^	1			
	SB	0.702^**^	0.775^**^	0.430^**^	0.976^**^	1		
	PT	-0.217^**^	0.013	-0.468^**^	-0.047	-0.072	1	
	PTP	-0.371^**^	-0.203^**^	-0.481^**^	-0.096	-0.037	0.529^**^	1
2016JZ	PV	1						
	TV	0.924^**^	1					
	BD	0.772^**^	0.470^**^	1				
	FV	0.894^**^	0.883^**^	0.597^**^	1			
	SB	0.794^**^	0.736^**^	0.609^**^	0.968^**^	1		
	PT	-0.122	0.14	-0.513^**^	-0.005	-0.082	1	
	PTP	-0.17	0.002	-0.381^**^	-0.108	-0.154	0.429^**^	1
2017JZ	PV	1						
	TV	0.835^**^	1					
	BD	0.829^**^	0.385^**^	1				
	FV	0.564^**^	0.744^**^	0. 190^**^	1			
	SB	0.362^**^	0.522^**^	0.277^**^	0.958^**^	1		
	PT	-0.351^**^	0.090	-0.680^**^	0.281^**^	0.320^**^	1	
	PTP	-0.277^**^	-0.015	-0.437^**^	0.020	0.032	0.461^**^	1

*PV* Peak viscosity, *TV* Trough viscosity, *FV* Final viscosity, *SB* Setback (FV − TV), *PT* Peak time, *PTP* Pasting temperature

^**^significant at *p* < 0.01, *significant at *p* < 0.05

**Fig. 2:**
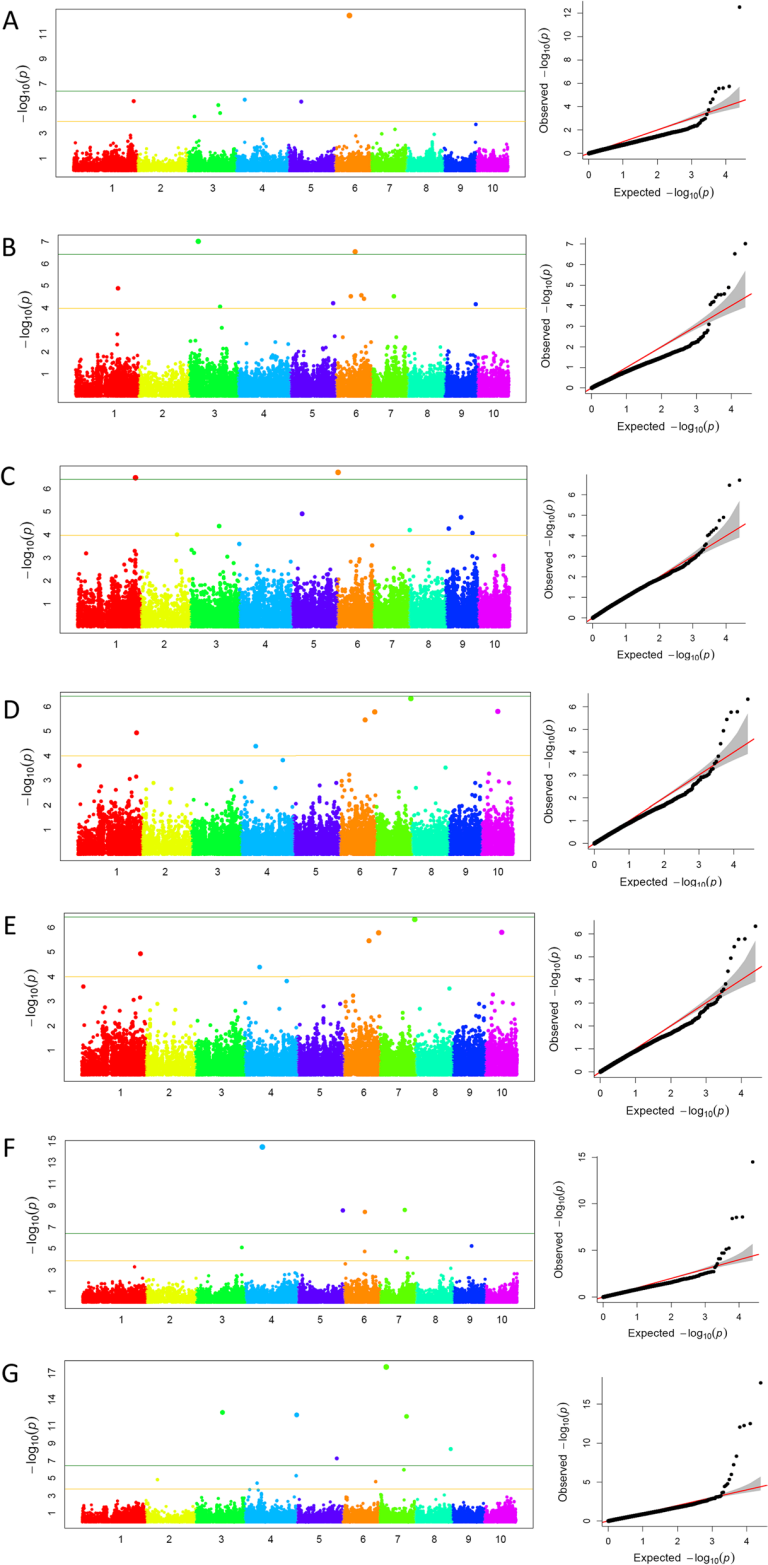
the manhattan polt and Q-Q polt of pasting properties by genome-wide association study. **A** peak viscosity; **B** trough viscosity; **C** breakdown; **D** final viscosity; **E** setback; **F** peak time; **G** pasting temperature

In order to find the SNPs that related to starch pasting properties, data of 25,331 SNPs and starch pasting properties were used based on the FarmCPU software. Significantly correlated SNPs were identified at the *P* < 10^–4^ level, and the candidate genes were identified (Fig. 2). A total of 48 SNPs correlated with pasting properties were detected in the four environments: 5, 7, 6, 9, 8, 8 and 5 SNPs for PV, TV, BD, FV, SB, PT and PTP, respectively. PZE_101122760, PZE_103046325, PZE_104089684, PZE_106039028, SYN26334 and PZE_110040421 were correlated with FV and SB; PZE_103091447 and PZE_105156016 were correlated with PV and TV; PZE_103096842 was correlated with PV and FV; and PZE_106067257 was correlated with TV and FV (Table 6).

**Table 6:** The SNPs associated with pasting properties (*P* < 10^–4^)

Trait	SNP	Chr	Genotype	Bin	Position	*P*-value	Environment
PV	SYN12772	3	A/G	3.03	35,084,281	4.40E-05	2016JZ
	PZE_103091447	3	G/T	3.05	147,223,326	5.26E-06	2016JZ
	PZE_103096842	3	A/G	3.05	155,433,336	2.35E-05	2016JZ
	PZE_105156016	5	A/C	5.06	204,313,319	5.36E-05	2015LY
	PZE_109088783	9	A/G	9.05	132,519,775	5.29E-05	2017JZ
TV	PZE_103091447	3	G/T	3.05	147,223,326	8.95E-05	2016JZ
	PZE_105155546	5	A/C	5.05	204,021,851	6.33E-05	2016JZ
	PZE_105156016	5	A/C	5.06	204,313,319	6.21E-05	2015LY
	PZE_106067257	6	C/T	6.04	120,189,163	2.74E-05	2016JZ
	PZE_108073083	8	C/T	8.05	126,033,112	1.11E-05	2017JZ
	PZE_108100984	8	C/T	8.06	155,557,132	3.44E-05	2017JZ
	PZE_109109322	9	C/T	9.06	145,602,633	6.84E-05	2016JZ
BD	PZE_101120543	1	G/T	1.05	148,525,339	8.89E-06	2016JZ
	PZE_103094159	3	A/C	3.05	151,148,617	8.56E-05	2016JZ
	PZE_103084229	3	C/T	3.05	135,898,087	4.29E-05	2017JZ
	PZE_105054501	5	G/T	5.03	50,733,228	1.25E-05	2017JZ
	SYN21058	6	A/G	6.00	3,779,374	1.96E-07	2017JZ
	SYN11767	9	A/C	9.02	14,438,563	2.06E-05	2016JZ
FV	PZE_101122760	1	C/T	1.05	153,387,202	5.75E-05	2015LY
	PZE_101147239	1	C/T	1.06	190,610,531	1.00E-06	2016JZ
	PZE_103046325	3	G/T	3.04	48,015,853	6.88E-05	2015LY
	PZE_103096842	3	A/G	3.05	155,433,336	1.72E-06	2016JZ
	PZE_104089684	4	A/G	4.06	164,750,722	4.08E-05	2015LY
	PZE_106039028	6	A/C	6.01	86,297,303	1.31E-06	2016JZ
	PZE_106067257	6	C/T	6.04	120,189,163	3.64E-06	2017JZ
	SYN26334	9	A/C	9.05	132,885,794	2.98E-05	2015LY
	PZE_110040421	10	A/C	10.03	77,111,930	1.65E-06	2017JZ
SB	PZE_101122760	1	C/T	1.05	153,387,202	5.95E-05	2015LY
	PZE_103046325	3	G/T	3.04	48,015,853	7.12E-05	2015LY
	PZE_104089684	4	A/G	4.06	164,750,722	5.71E-05	2015LY
	PZE_104099048	4	C/T	4.07	174,469,971	1.23E-06	2016JZ
	PZE_106039028	6	A/C	6.01	86,297,303	7.74E-06	2016JZ
	PZE_107043379	7	C/T	7.02	74,685,305	2.88E-05	2016JZ
	SYN26334	9	A/C	9.05	132,885,794	3.43E-05	2015LY
	PZE_110040421	10	A/C	10.03	77,111,930	6.09E-06	2017JZ
PT	SYN9068	1	A/C	1.07	210,940,058	2.56E-05	2015QZ
	SYN33389	3	A/C	3.08	213,893,310	7.71E-06	2016JZ
	PZE_104099048	4	C/T	4.07	174,469,971	3.86E-05	2015LY
	PZE_106049618	6	C/T	6.03	98,823,263	3.78E-09	2016JZ
	PZE_107060445	7	C/T	7.02	110,670,176	6.58E-06	2017JZ
	PZE_107060447	7	A/C	7.02	110,670,253	2.86E-05	2017JZ
	SYN32385	7	G/T	7.03	129,793,852	7.70E-05	2016JZ
	SYN32384	7	A/G	7.03	129,793,957	7.70E-05	2016JZ
PTP	PZE_104039782	4	C/T	4.05	59,365,625	3.71E-05	2017JZ
	PZE_105099535	5	C/G	5.04	146,962,269	8.28E-05	2015LY
	PZE_106103881	6	A/G	6.06	155,397,283	2.64E-05	2017JZ
	PZE_108031001	8	C/G	8.03	35,001,109	2.41E-05	2015LY
	PZE_109054108	9	G/T	9.03	90,505,476	3.77E-06	2015LY

*PV* Peak viscosity, *TV* Trough viscosity, *FV* Final viscosity, *SB* Setback (FV − TV), *PT* Peak time, *PTP* Pasting temperature

### GO analysis of candidate genes

**Fig. 3:**
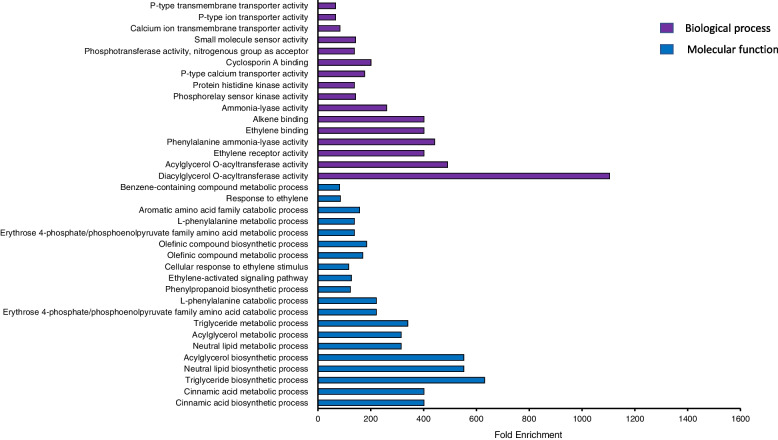
Gene ontology (GO) enriched terms associated with differentially expressed genes (DEGs) in starch content

**Fig. 4:**
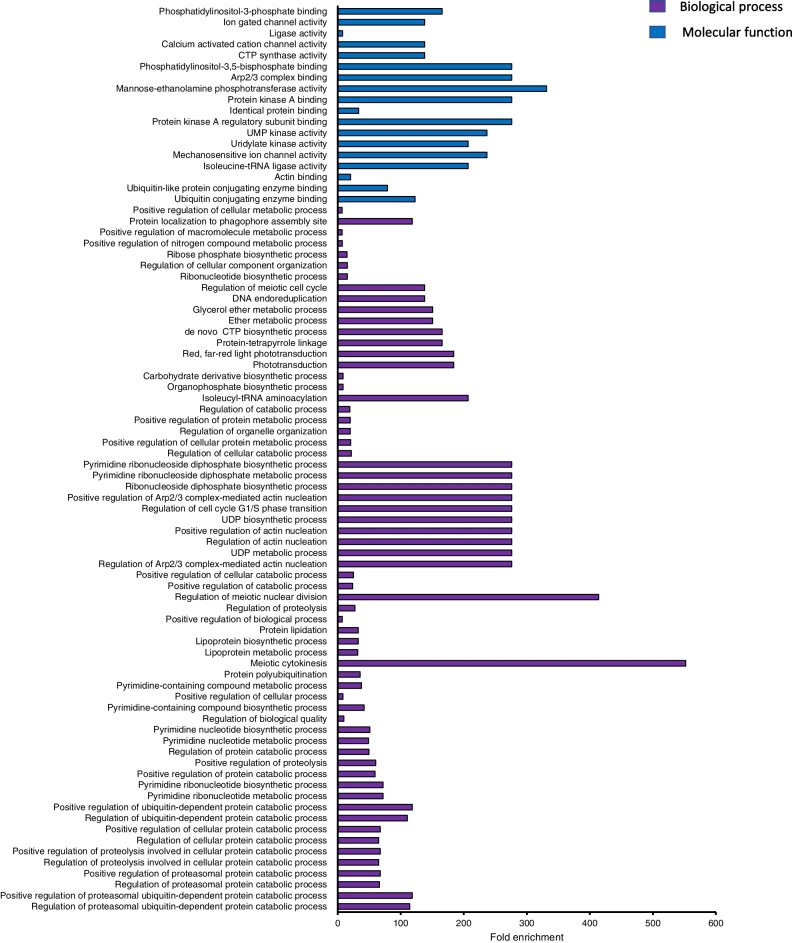
Gene ontology (GO) enriched terms associated with differentially expressed genes (DEGs) in starch pasting properties

Based on the genome-wide association study results, 26 and 37 candidate genes were found to be related to starch content and starch pasting properties respectively (Tables 7 and 8). In order to gain insights into the functions of the identified candidate genes, Gene Ontology term enrichment analysis was performed through ShinyGO database [17]. For starch content, the annotated results were classified into two parts: biological process (16 categories) and molecular function (20 categories) (Fig. 3). The results showed that, in biological process, the fold enrichment of triglyceride biosynthetic process, neutral lipid biosynthetic process, acylglycerol biosynthetic process reach to 631, 553, 552 respectively. In addition, the diacylglycerol O-acyltransferase activity (the fold enrichment reached to 1104) was one of the most enriched categories of molecular function. For starch pasting properties, 64 biological process related categories and 18 molecular function related categories were identified (Fig. 4). In biological process, the fold enrichment of positive regulation of biological process, positive regulation of cellular process, positive regulation of cellular metabolic process, positive regulation of nitrogen compound metabolic process is 816, 900, 711, 691 respectively. Moreover, the fold enrichment of ligase activity, actin binding, identical protein binding is 642, 164, 99 respectively in molecular function.

**Table 7:** Information of candidate gene associated with starch content

SNP	Candidate gene	Candidate gene function
PZE_101052003	LOC103633789	rho GTPase-activating protein 7
PZE_101130213	GRMZM2G142660	SPX and EXS domain-containing protein 1
PZE_101183173	GRMZM5G870629	ABC transporter G family member 11
PZE_101222418	GRMZM2G147917	glycerol-3-phosphate 2-O-acyltransferase 6
PZE_103020134	GRMZM2G010693	Protein NSP-INTERACTING KINASE 3
PZE_103073639	GRMZM2G114924	S-adenosyl-L-methionine-dependent Methyltransferase super family protein
PZE_104013159	GRMZM2G008259	PHD finger protein
PZE_104050777	GRMZM2G445478	hypothetical protein
PZE_104099386	GRMZM2G472023	Protein TONSOKU
PZE_105024467	GRMZM2G102601	ethylene receptor1-25
PZE_105063750	GRMZM2G088212	catalase 1
SYN37380	GRMZM2G011631	DNA polymerase epsilon catalytic subunit A
PZE_105099535	GRMZM2G138423	ADP,ATP carrier protein
PZE_105086878	GRMZM2G006107	Peptidyl-prolylcis-trans isomerase CYP95
PZE_105129479	GRMZM2G029048	phenylalanine ammonia lyase9
SYN3414	GRMZM2G066400	WPP domain-associated protein
PZE_106049999	GRMZM2G112337	microtubule-associated protein MAP65-1a
PZE_106054189	GRMZM2G169089	diacylglycerol O-acyltransferase 1
PZE_106067078	GRMZM2G162783	UDP-glycosyltransferase 708A6
PZE_106054189	GRMZM2G169089	diacylglycerol O-acyltransferase 1
PZE_107065016	GRMZM2G004207	serine/threonine protein kinase
PZE_108005661	GRMZM2G155260	receptor-like protein kinase ANXUR1
PZE_108028552	GRMZM2G001421	Indole-3-acetic acid amido synthetase GH3.6
PZE_108135907	GRMZM2G111354	WRKY transcription factor 75
PZE_110013629	GRMZM2G001421	cytochrome P450 71A1
PZE_110093311	GRMZM2G391042	calcium-transporting ATPase 8

**Table 8:** Information of candidate gene associated with pasting properties

Trait	SNP	Candidate gene	Candidate gene function
PV	SYN12772	GRMZM2G314563	GPI ethanolamine phosphate transferase 3
	PZE_103091447	GRMZM2G361501	junction endonuclease MUS81
	PZE_105156016	GRMZM2G357923	ATP-dependent RNA helicase DEAH13
	PZE_109088783	GRMZM2G007514	protein SCAR2
TV	PZE_103091447	GRMZM2G361501	junction endonuclease MUS81
	PZE_105155546	GRMZM2G090609	caleosin related protein
	PZE_105156016	GRMZM2G357923	ATP-dependent RNA helicase DEAH13
	PZE_106067257	GRMZM2G333923	DUF679 domain membrane protein 7
	PZE_108073083	GRMZM2G090963	ATP-dependent DNA helicase Q-like SIM
	PZE_108100984	GRMZM2G149535	Amino acid kinase family protein
	PZE_109109322	GRMZM2G028643	serine/threonine-protein kinase At2g14440
BD	PZE_101120543	GRMZM2G135045	Xaa-Pro aminopeptidase 3
	PZE_103094159	GRMZM2G146280	phosphoinositide-interacting protein 3
	PZE_103084229	GRMZM2G046610	methyltransferase PMT27
	PZE_105054501	GRMZM2G177535	tyrosine-sulfated glycopeptide receptor 1
	SYN21058	GRMZM2G143646	pentatricopeptide repeat-containing protein
	SYN11767	GRMZM2G000423	2-oxoglutarate (2OG) and Fe(II) dependent oxygenase super family protein
FV	PZE_101147239	GRMZM2G166407	pentatricopeptide repeat-containing protein
	PZE_104089684	GRMZM2G122843	signal peptide peptidase family protein
	PZE_106039028	GRMZM2G416386	aminodeoxychorismate synthase
	PZE_106067257	GRMZM2G333923	DUF679 domain membrane protein 7
	SYN26334	LOC103639262	rab3 GTPase-activating protein catalytic subunit
SB	PZE_101122760	LOC103639905	pectinesterase 11
	PZE_104089684	GRMZM2G122843	signal peptide peptidase family protein
	PZE_106039028	GRMZM2G416386	aminodeoxychorismate synthase
	PZE_107043379	GRMZM2G363052	AP2/EREBP transcription factor super family protein
	SYN26334	LOC103639262	rab3 GTPase-activating protein catalytic subunit
PT	SYN9068	GRMZM2G131205	cinnamoyl CoA reductase 1
	SYN33389	GRMZM2G121878	carbonic anhydrase
	PZE_106049618	GRMZM2G074438	transcription factor bHLH48
	PZE_107060445	GRMZM2G066171	RING zinc finger domain super family protein
	SYN32385	GRMZM2G059225	ADP-ribosylation factor GTPase-activating protein AGD3
PTP	PZE_104039782	GRMZM2G085000	Thioredoxin family protein
	PZE_105099535	GRMZM2G138423	ADP,ATP carrier protein
	PZE_106103881	GRMZM2G158526	centromeric histone H3
	PZE_108031001	GRMZM2G331566	endoglucanase 1
	PZE_109054108	GRMZM2G348666	Isoleucine–tRNA ligase cytoplasmic

*PV* Peak viscosity, *TV* Trough viscosity, *FV* Final viscosity, *SB* Setback (FV − TV), *PT* Peak time, *PTP* pasting temperature

## Discussion

Starch or amylum is a polymeric carbohydrate consisting of numerous glucose units joined by glycosidic bonds [18]. This polysaccharide is produced by most plants for energy storage. In plants, the extra glucose is changed into starch which is more complex than the glucose produced by plants. Starch biosynthesis is a complex process in plants. Starch is produced by first converting glucose 1-phosphate to ADP-glucose using the enzyme glucose-1-phosphate adenylyltransferase in plant. The starch synthase then adds the ADP-glucose via a 1,4-alpha glycosidic bond to a growing chain of glucose residues, liberating ADP and creating starch. Starch content in maize kernels is a complex process [19]. In this study, the heritability of starch, protein and oil content were 85.82%, 82.73% and 80.69% respectively. It indicates the important role of genotypes in expression of traits and maize breeding. Identification of the key genes related to the variation in starch content and pasting properties can help to understand the genetic background of starch quantity and maize kernels quality and expand its application. In addition, the starch content and pasting properties SNPs we found in this study can provide some useful markers for maize marker-assisted selection.

In this study, we identified 37 SNPs and 26 candidate genes for starch content through GWAS analysis in the 292 inbred lines. In addition, 48 SNPs correlated with pasting properties were detected. The GO analysis indicated that some carbohydrate metabolism related processes, such as triglyceride, neutral, acylglycerol biosynthetic process, have an important influence on starch content. Consistent with previous studies, many carbohydrate metabolism related QTLs or genes participate in starch metabolism [20–23].

When we consider the genes identified here and previously identified QTLs or genes for starch content [7, 8, 21, 23–28], we note that the identified starch content related genes by different studies are different. This finding could be the result of differences in population size, genetic backgrounds, statistical analysis methods, environmental effects, etc. In addition, some auxin related genes were detected in this study, such as Indole-3-acetic acid amido synthetase GH3.6 [29], rho GTPase-activating protein [30], in accordance with the previous studies that auxin participates in the starch metabolism [31, 32]. These finding indicated a complex regulation network related to starch content, and the starch content could be regulated be different genes under different environments.

In order to investigate the molecular mechanism of starch pasting properties in maize, we further identified locations of associated SNPs for possible candidate genes. In this study, we identified 48 SNPs and 37 genes that correlated with starch pasting properties. According to functional annotations, these candidate genes were primarily categorized in various biological process and molecular function, such as positive regulation of cellular process, positive regulation of cellular metabolic process, positive regulation of nitrogen compound metabolic process, ligase activity, actin binding, identical protein binding etc. The transcription factors included AP2/EREBP, NAC were detected in this study. Some of the candidate genes or their homologous genes are known genes linked to carbohydrate metabolism. For example, ZmNAC34, a maize NAC transcription factor, negatively regulates starch synthesis in rice [33]. WRINKLED1 (WRI1) belongs to AP2/EREBP transcription factor. Its function in dicots for fatty acids synthesis [34].

## Conclusions

Our study provides an important extension of maize starch metabolism and starch pasting properties. As a result, 26 and 37 candidate genes were found to be related to starch content and starch pasting properties respectively, indicated a complex regulatory network about regulation of starch content and starch pasting properties in maize. It also indicated that the regulatory network of starch content and starch pasting properties could be different between different environment conditions. This finding reflects the complex nature of maize starch metabolism, which depends on a large number of different environment related genes.

## Materials and methods

### Plant material and field design

A population composed of 292 maize inbred lines (The maize inbred line were obtained from Qingdao Agricultural University, Table 9) belonging to four subgroups (Lancaster, Lvdahonggu, P group, and Sipingtou) was used for GWAS. The 292 maize inbred lines were grown in three replications at four locations in China, 2015Qingzhou (Shandong Province, 2015QZ), 2015Luoyang (Henan Province, 2015LY) and Jiaozhou (Shandong Province) in 2016 and 2017 (2016JZ and 2017JZ). The materials were arranged in a randomized complete block design, and each inbred line was grown in a single row measuring 3 m in length and 0.6 m in width, with 15 individual plants per row. Five to eight plants in each row were self-pollinated when more than 80% silk appeared. After maturity, the ears were harvested and naturally dried. The dried ears (water content < 14%) of each plot were shelled manually and bulked for kernel composition trait tests. Pasting properties were measured using a Rapid Visco Analyzer (RVA, Model 3D, Perten, Sweden) and analyzed using Thermal Cycle for Windows (TCW) software. The sample suspension of each inbred line was incubated at 50 °C for 1 min; the temperature was increased to 95 °C, maintained for 2.5 min, and finally cooled to 50 °C and maintained for 1 min. Three primary RVA parameters, peak viscosity (PV), trough viscosity (TV), and final viscosity (FV), were obtained from the pasting curve. Two secondary RVA parameters, breakdown (BD = PV − TV) and setback (SB = FV − TV), were calculated from the primary parameters. Peak time (PT) and pasting temperature (PTP) were also recorded. Trait measurements averaged over the three replications were used as the preliminary data.

**Table 9:** List of 292 maize inbred lines

Number	Name	Number	Name	Number	Name	Number	Name
1	Ye478	74	LY023	147	L219	220	Qi205
2	Zao49	75	LY024	148	L502-196	221	Qi318
3	E28	76	LY025	149	LD3162	222	107X
4	B73	77	LY026	150	LX9801	223	11N597
5	444	78	LY028	151	M01	224	129–2405
6	P138	79	LY029	152	M4	225	ReBS11
7	P170	80	LY030	153	MJ02	226	Renbai
8	853	81	LY031	154	ML-1	227	Santuan
9	7922	82	LY032	155	zm5536	228	Shannong206a
10	81,162	83	LY033	156	zm5537	229	Shannong206b
11	K12	84	LY034	157	zm5538	230	Shan814
12	Hainan1/6	85	LY035	158	zm5539	231	Shen137
13	Ji846	86	LY036	159	zm5540	232	Shendan16F
14	Qi319	87	LY037	160	zm5541	233	Shengyu88m
15	Qingnong105F	88	LY038	161	zm5542	234	Shunyao7hao
16	Qingnong105M	89	LY039	162	zm5543	235	Sun1
17	JingD24	90	LY042	163	Ao20-3	236	Sun2
18	Liangyu88m	91	LY044	164	o894-2	237	Sun3
19	13tian-3	92	LY045	165	Bai515	238	Tian06-261
20	319B	93	LY046	166	Bai515	239	Tian16
21	335Xuan	94	LY047	167	Bao-1	240	Tian-2
22	340G	95	LY049	168	Benyu15	241	Zhong102
23	414Xi	96	LY050	169	Chang7-2G	242	Zhongdan909X
24	496WP	97	LY054	170	Chao6X	243	Zhongxi091
25	78599Xuan	98	K12HF304	171	Zhaobai-1	244	Zhunuo-7
26	LY055	99	K12HF76	172	Chong17-2	245	Zyao515
27	LY056	100	K36	173	Chong17-2	246	92Huang40
28	LY057	101	K6H4057	174	Dan340	247	A632
29	87–20	102	K6H6079	175	Dan638	248	AMD43X
30	K12-452	103	K6H6179	176	Dan638	249	FeiLB-2
31	K12-512	104	K6H6784	177	Dan998	250	Nonghua101m
32	K12-526	105	K6H9103	178	Dansy 3–1	251	Nnuo-2
33	K12-76	106	K8112	179	Danhuang25	252	Nuo-3
34	K12HF184	107	KN-1	180	Du6607	253	Nuo-4
35	K910G	108	KN-1 m	181	FeiLB-1	254	Nuo-5
36	KHL88	109	LY059	182	WYH-2	255	Pengtian11-A
37	CML13	110	LY060	183	WYH-3	256	Xi1-4
38	CML84	111	LY061	184	X178	257	Xia514
39	CML99	112	LY062	185	XD28	258	Xia844
40	CML199	113	LY064	186	Y53	259	Xia987
41	CML255	114	LY065	187	YM-8	260	Xia996
42	CML299	115	LY066	188	YWH67	261	Xian96
43	CML306	116	LY068	189	zm5535	262	XianfengXuan
44	CML385	117	LY069	190	FeiLB-3	263	Xianyu698X
45	D811	118	LY070	191	Feng273	264	Xin1391
46	DH7823	119	LY071	192	Fu96	265	XinDH
47	Ex	120	LY073	193	HaiY18	266	Yan103
48	LY001	121	LY074	194	Hainan-2	267	Yan172
49	LY002	122	LY11-11	195	Hua160	268	254
50	LY006	123	LYM1	196	HuayuW13	269	Yi67
51	LY007	124	LYM2	197	Huang5	270	Zao10
52	LY009	125	LYM3	198	Huang515	271	Zao10
53	LY010	126	LYM4	199	Jichu1	272	Zhao835
54	LY013	127	K_12_-146	200	107–8	273	Zheng0510
55	LY014	128	K_12_-148	201	jiM67	274	Zheng58G
56	LY016	129	K_12_-160	202	Jizao48	275	H231
57	LY017	130	K1_2_-176	203	Jizaobai	276	H90
58	LY018	131	K_12_-272	204	Jinhai5	277	189
59	LY019	132	K_12_-386	205	13H-342	278	BC2433
60	LY020	133	HO-3–4	206	13H-375	279	BM
61	LY021	134	NF358	207	Liao3162	280	230
62	JH271	135	NLEBM-4	208	Liao3180	281	D729
63	K12B	136	OA1207	209	liaoyu20mu	282	244
64	H901	137	P1211-6	210	Lu65	283	FC521
65	ML-3	138	488	211	Mei24242	284	FR218
66	MLBJ	139	785	212	Mei338	285	ML-2
67	MQ-3	140	S122	213	Meikang-1	286	Tie9010
68	MY-4	141	T123	214	Meikang-2	287	Tie98042
69	MY6-8	142	T29803	215	Meikang-3	288	TieX98042
70	NF-35	143	W42-2	216	Meikang-4	289	Tiedan9010
71	TW263	144	WL	217	Meixuan	290	Weihaibai
72	TW623	145	L01125	218	Tian-4	291	Wei1122
73	LY022	146	L0167	219	Pengtian33-A	292	Xixingnuo-6

### Analysis of phenotypic data

All analyses were performed using the statistical analysis software package IBM SPSS Statistics 20.0. The broad-sense heritability (H^2^) was calculated as follows: ${\mathrm{H}}^{2}={\upsigma }_{\mathrm{g}}^{2}/({\upsigma }_{\mathrm{g}}^{2}+{\upsigma }_{\mathrm{gl}}^{2}/\mathrm{n}+{\upsigma }_{\mathrm{e}}^{2}/\mathrm{nr})$, where ${\upsigma }_{\mathrm{g}}^{2}$, ${\upsigma }_{\mathrm{gl}}^{2}$ and ${\upsigma }_{\mathrm{e}}^{2}$ were estimates of genotype, genotype environment interaction and experimental error variances, while n and r were the numbers of environments and replications, respectively [35].

### DNA Extraction and SNP Genotyping

DNA for SNP genotyping was extracted from a seeding of each line by the CTAB method [36]. A total of 55,126 SNPs were selected from the whole maize genome and genotyped with the MaizeSNP50 BeadChip from Pioneer DuPont (U.S). The 25,331 SNPs remaining after excluding SNPs with a missing rate > 20%, heterozygosity > 10% and minor allele frequency (MAF) < 0.05 were used for GWAS.

### Association analysis

The SNPs from 292 inbred lines were analyzed with the FarmCPU (Fixed and Random Model Circulating Probability Unification), which used a Fixed Effect Model (FEM) and a Random Effect Model (REM) alternately. The source code of the algorithm (http://zzlab.net/FarmCPU/FarmCPU_functions.txt) was invoked through the R software GAPIT package [Zhu et al. 2018]. The population structure was assessed with unlinked markers (*r*^*2*^ = 0.1) using STRUCTU RE ver. 2.3.4 [37], based on the highest delta K value representing genetic clusters [38].

### Candidate genes analysis

Based on the results, SNPs associated with starch pasting properties were identified. In this study, the genome from maize line B73 was used as the reference genome for candidate gene analysis [39, 40]. The genes’ p ‘’ositions and functions were annotated according to MaizeGDB database (http://www.maizegdb.org/)(references) and NCBI database (http://www.ncbi.nlm.nih.gov/)(references). The ShinyGO database (http://bioinformatics.sdstate.edu/go/) was used to GO analysis of the candidate genes [17].

## Data Availability

The datasets generated and/or analyzed during the current study are available in the Figshare repository, https://doi.org/10.6084/m9.figshare.20347005.v1.
